# Ultrasmall Cyclodextrin‐Based Nanogels as Drug Delivery Systems

**DOI:** 10.1002/open.70274

**Published:** 2026-08-04

**Authors:** Andrea Cesari, Simona Braccini, Maria Antonietta Casulli, Kento Ishigaki, Takeshi Hashimoto, Takashi Hayashita, Dario Puppi, Fabio Bellina

**Affiliations:** ^1^ Department of Chemistry and industrial Chemistry University of Pisa Pisa Italy; ^2^ Micro and Nano Systems (MNS) Leuven (Arenberg) Leuven Belgium; ^3^ Graduate School of Science and Technology Department of Materials and Life Sciences Faculty of Science and Technology Sophia University Tokyo Japan

**Keywords:** combretastatin A‐4, cyclodextrin, drug delivery systems, osthole, ultrasmall nanogel

## Abstract

Ultrasmall cyclodextrin‐based nanogels (CDngs) crosslinked with ethylene glycol diglycidyl ether (EGDE) were tested as drug delivery systems for poorly water‐soluble anticancer agents for the first time. Two compounds with well‐established pharmacological activity but critical solubility limitations, i.e., Osthole and Combretastatin A‐4 (CA‐4), were selected to evaluate the encapsulation capacity and performance of the nanogel platform. After preliminary screening through experimental and docking investigation, CA‐4 was chosen as the reference compound. Among the formulations developed, the CA‐4/loaded nanogel displayed the highest encapsulation efficiency (EE%). Despite a less‐than‐ideal host–guest fit, the large cavity of γ‐CD allows for efficient drug encapsulation and higher loading capacity. The optimized formulation was tested in vitro to assess both the biocompatibility of γ‐CDngs, as well as the anticancer efficacy of CA‐4 loaded γ‐CD and γ‐CDngs. We demonstrate that empty systems exhibit intrinsic biocompatibility, showing no cytotoxicity toward the A2780 cancer cell line under experimental conditions. Furthermore, the CA‐4 loaded γ‐CDngs retained and enhanced the cytotoxic properties of the free drug against this cell line, compared to the corresponding native γ‐CD. Collectively, these findings validate CDngs as a promising and innovative platform for the delivery of hydrophobic anticancer molecules and support their potential for further therapeutic development.

## Graphical abstract

Cyclodextrin (CD) nanogels provide a highly effective, biocompatible platform for advanced drug delivery. These specialized networks successfully encapsulate active ingredients, significantly improving overall drug solubility. Furthermore, the resulting nanogels exhibit enhanced toxicity toward cancer cell lines compared to the parent CDs.

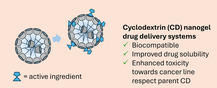

## 1 Introduction

**FIGURE 1:**
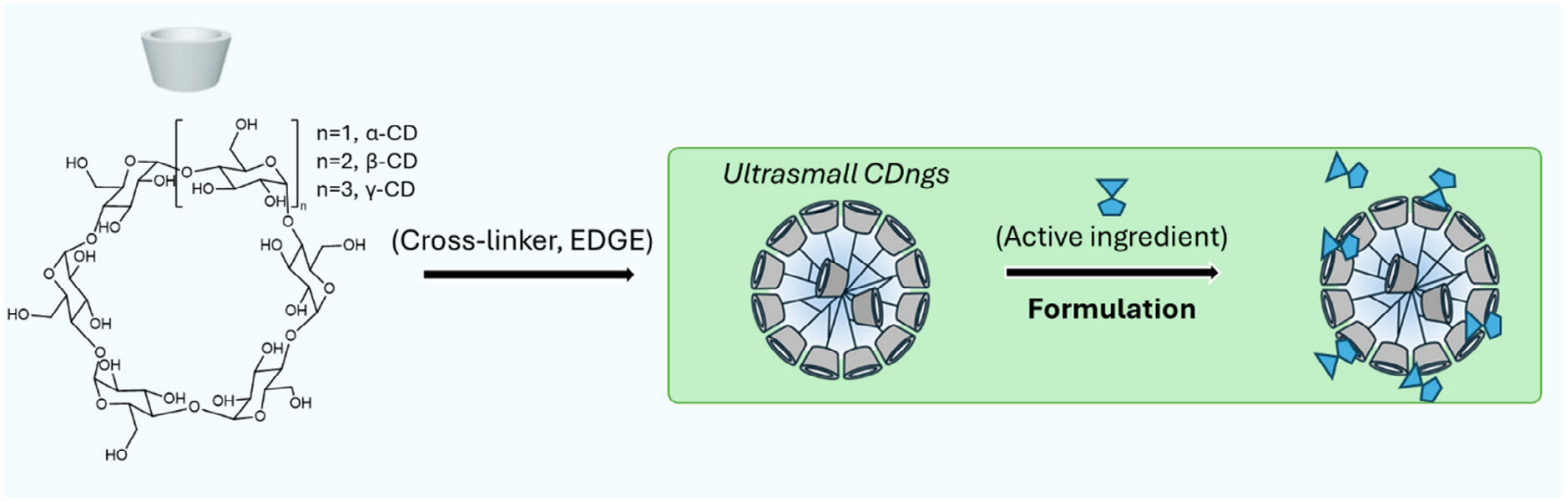
Schematic preparation of ultrasmall CDngs from native CDs and consequent drug loading.

The development of innovative drug delivery systems (DDSs) capable of enhancing the solubility, stability, and bioavailability of poorly water‐soluble therapeutics continues to represent a significant challenge in contemporary pharmacology and nanomedicine [1]. Many phytochemicals, although mechanistically promising, suffer from critical biopharmaceutical limitations such as poor aqueous solubility, rapid degradation, and limited systemic availability, all of which hinder their clinical translation [2]. Overcoming these intrinsic physicochemical barriers requires advanced DDSs that can both improve solubilization and enable controlled and targeted release. Among the widely used systems for DDS development, cyclodextrins (CDs) feature a unique toroidal geometry (constructed from α‐1,4‐linked glucose units) composed of a hydrophobic inner cavity and a hydrophilic outer surface (Figure 1). This amphiphilic structural arrangement enables the formation of supramolecular inclusion complexes, which encapsulate hydrophobic drug molecules, thereby enhancing their aqueous solubility, protecting them from environmental degradation, and modulating their release characteristics [3, 4, 5]. The three principal native forms, α‐, β‐, and γ‐CD, constituted by 6, 7, and 8 glucose units, respectively, have been widely exploited in pharmaceutical formulation due to their biocompatibility, biodegradability, and regulatory acceptance, as well as their capacity to alter the physicochemical behavior of guest molecules [6]. Besides their simple use as functional excipients for poorly soluble molecules [7] or as active principles themselves (i.e., sugammadex) [8], CDs and their chemically derivatized substrates have been employed for the production of more complex DDSs. As a matter of fact, CD‐based DDSs have flourished over the past few years. As an example, CDs have been incorporated into nanosized liposome hybrid systems [9], or used as building blocks for the production of crosslinked polymers [10, 11, 12] and nanosponges [13]. Of considerable interest are also their stimuli‐responsive implementation, such as mesoporous silica nanoparticles‐CD system (light responsive) [14] or thiolated‐CD systems (redox responsive) [15]. However, these CD‐based carriers generally exhibit limitations that impede effective drug delivery in vivo. CD‐liposome hybrids and polymers may suffer from relatively large and heterogeneous particle sizes, limited drug loading capacity compared to their size, uncontrolled or slow release kinetics, and stability challenges in complex biological environments, all of which can restrict tissue penetration and therapeutic efficacy. Nanosponges, in particular, can form densely crosslinked networks that hinder drug access and release, and their larger particle sizes may reduce bioavailability and tumor accumulation [16]. On the other hand, nanogels combine the advantages of hydrogel carriers with nanoscale delivery, enabling enhanced tissue permeation, improved cellular uptake, and better biodistribution [17]. The smaller size and soft, hydrated network of nanogels can decrease rapid clearance by the mononuclear phagocyte system, facilitate deeper extravasation into target tissues, and allow for adjustable drug release profiles [18]. Specifically, CD‐based nanogels (CDngs) inherit the complexation properties of native CDs [17]. Recently, our group has developed a controlled synthesis of ultrasmall CDngs (10–25 nm in diameter) by crosslinking ethylene glycol diglycidyl ether (EGDE) within a surfactant‐assisted environment [19]. CDngs have emerged as interesting electrochemical and NMR sensors for curcumin detection, owing to their porosity, physical stability, high water content, and extensive binding capacity toward hydrophobic molecules [20]. Hydrophobic molecules can be noncovalently included within the CD cavities while simultaneously being stabilized within hydrophobic microdomains of the nanogel (Figure 1). New developments have been explored using CDngs as nitric oxide photoreleasing agents [21] and imaging tracers [17], suggesting their versatility in biological environments.

In view of these considerations, we present here a thorough analysis of ultrasmall CDngs for use as a DDS. We assess their feasibility using two hydrophobic active principles, rationalize their interaction with NMR spectroscopy and docking analysis, and evaluate the selected formulation against the A2780 cancer cell line (human ovarian cancer).

## 2 Materials and Methods

### 2.1 Materials

Osthole (OST) was purchased from Zentek srl (Milan, Italy). *cis*‐Combretastatin A‐4 (CA‐4) was synthesized by following a two‐step literature procedure, obtaining the desired product with a 98% GLC purity and a 40% yield [22]. α‐CD, β‐CD, and γ‐CD were purchased from CarboHyde (Budapest, Hungary). Deuterated water (D_2_O, 99.9%) and deuterated DMSO (DMSO‐d_6_, 99.8%) were purchased from Deutero GmbH (Kastellaun, Germany). Sodium hydroxide (NaOH), toluene, EGDE, 1‐hexanol, and propranolol hydrochloride were obtained as special‐grade reagents from Fujifilm Wako Pure Chemical Corp. (Osaka, Japan). [Dilauryl(dimethyl)ammonium]bromide (DDAB) was purchased from Tokyo Chemical Industry Co., Ltd. (Tokyo, Japan). All other organic solvents and reagents were commercially available with guaranteed grade and used as received. Water was doubly purified by a prepurification system (WG222, Yamato Scientific Co., Ltd. Tokyo, or Elix‐ Essential 5, Merck Millipore, MA, USA) and a Milli‐Q water system (Milli‐Q Advantage, Merck Millipore, MA, USA) before use.

### 2.2 Cell Culture

A2780 human ovarian cancer cell line was purchased from the European Collection of Authenticated Cell Cultures (ECACC, Salisbury, UK; Catalog no. 93112519). Cells were cultured according to the manufacturer's instructions using Roswell Park Memorial Institute (RPMI) 1640 medium (Merck, Milan, Italy) supplemented with 10% fetal bovine serum (FBS; Merck, Milan, Italy), 1% penicillin/streptomycin (10 000 U/mL:10 mg/mL; Merck, Milan, Italy), 2 mM l‐glutamine (Merck, Milan, Italy), and an antimycotic (Plasmocin; InvivoGen, Toulouse, France), at 37 °C in a 5% CO_2_‐enriched atmosphere.

### 2.3 Instruments

Dynamic light scattering (DLS) measurements were conducted using Zetasizer Nano ZS (Malvern Instruments Ltd., Malvern, Worcestershire, United Kingdom) at room temperature (RT) in water with each type of CDngs at 3.0 mg/mL. For the DLS measurements, quartz cells (DTS 2145 and ZEN 2112) were used. NMR spectra were obtained using a JEOL ECZR 500 MHZ with a probe operating at 500 MHz. Samples were vortexed using a Velp Scientifica TX4 mixer and sonicated in an Elmasonic S40H when necessary, to homogenize the solutions. NMR samples containing CDngs were prepared from stock solutions of the corresponding CDngs in deuterated water (15 mg/mL).

### 2.4 Docking

For docking analysis, a preliminary global minimum geometry optimization of the ligands was conducted using the ORCA quantum chemistry software package [23, 24] via the Global Optimizer Algorithm (GOAT) [25]. The global minimum conformation for OST and CA‐4 is reported in Figures S1 and S2 of the Supporting Information. The initial structures of the CDs were obtained from CSD crystallographic data (α‐CD [26], β‐CD [27], and γ‐CD [28]), cleaned of ions and ligands, and finally geometry optimized with Avogadro 1.2.0. Docking was conducted using Autodock Vina [29] (exhaustiveness = 64, num_modes = 20, energy_range = 5).

### 2.5 CDngs Synthesis

For the synthesis of CDngs, more details are reported in our previous work [19]. Briefly, 0.33 mmol of native β‐CDs (or γ‐CDs) was diluted in 5 mL of 0.2 M NaOH. In another beaker, 0.143 mmol of DDAB was diluted in 14.3 mL of toluene. A 3 mL volume of EGDE cross‐linker was added to the alkaline aqueous solution containing the native β‐CDs (or γ‐CDs). After sonication, 2.5 mL of 1‐hexanol was added very slowly. Afterward, toluene containing DDAB was added to the β‐CDs (or γ‐CDs) alkaline aqueous solution. After 15 min of standing, homogenization of the solution was performed for 10 min (homogenizer setting parameters: output control 2.5 (15 W); duty cycle 50). Afterward, the solution was left stirring at RT for 27 h. The aqueous phase was collected, assuming the partition of nanogels into this layer, and 20 mL of acetone was added to the aqueous phase. By performing a centrifugation (10 000 rpm for 10 min), the toluene and the surfactant residuals moved to the acetone part. Afterward, the pH was adjusted from basic to a neutral condition, through the addition of hydrochloric acid (HCl). Dialysis was carried out in Milli‐Q water for 7 days. Filtration was achieved in two steps: the first used a paper filter with 1 μm pores and, subsequently, a membrane filter with 50 nm pores. After freeze‐drying, β‐CDngs (or γ‐CDngs) were obtained and diluted to 50 mg/mL in Milli‐Q water.

### 2.6 Formulations

Formulation for the preparation of loaded CD or CDngs was conducted as follows: the active ingredient, selected at a theoretical final concentration of 1 mM, was dispersed in CDngs stock solution (300 μL), sonicated for 10 min, vortexed for 4 h (800 rpm), and then centrifuged (30 min/4000 rpm). The encapsulation efficiency (EE%) was calculated using NMR spectroscopy in quantitative conditions with a DMSO external standard for OST and CA‐4. The EE% was determined as the ratio of the quantified drug amount to the total amount of drug added, expressed as a percentage. Drug Loading (DL%) was calculated as the ratio of the mass of the drug entrapped within the CDngs to the total mass of the formulation (drug plus CDngs), expressed as a percentage. The concentration of active ingredients was quantified by NMR spectroscopy in fresh samples, just before the cell line tests.

### 2.7 IC_50_ Studies

A2780 cells were seeded in 96‐well plates at a density of 3 × 10^3^ cells/well and incubated for 24 h at 37 °C in 5% CO_2_ before exposure to CA‐4 and CA‐4/γ‐CD (or CA‐4/γ‐CDngs) at a concentration range of 0.39 to 25 nM. CA‐4 was prepared as a 1 mM stock solution in DMSO and diluted in growth medium to a final DMSO concentration of ≤0.0025% (v/v), while the drug‐loaded γ‐CD or γ‐CDngs water solutions were diluted in cell culture medium to a maximum water concentration of 0.05% (v/v). To evaluate the potential cytotoxicity of the carrier, cells were exposed to unloaded γ‐CD or γ‐CDngs, diluted in growth medium under the same experimental conditions used for the drug‐loaded formulations (0.05–3.13 µg/mL). Cells grown in cell culture medium with DMSO 0.0025% (v/v) or water 0.05% (v/v) were used as controls for CA‐4 and drug‐loaded/unloaded γ‐CD or γ‐CDngs systems, respectively. After 72 h of treatment, WST‐1 assay (Merck, Milan, Italy) was used to assess cell viability. Cells were treated with the tetrazolium salt reagent (1:10 in growth medium) for 4 h. Subsequently, absorbance was recorded at 450 and 655 nm, as a reference, using a microplate reader (Bio‐Rad, Milan, Italy). The 50% inhibitory concentration (IC_50_) was calculated via nonlinear regression analysis using a sigmoidal dose–response fit (GraphPad Prism, La Jolla, CA). Results were expressed as the mean ± standard deviation of three independent experiments.

### 2.8 Statistical Analysis

Data are expressed as the mean ± standard deviation. Statistical significance was assessed using one‐way analysis of variance (ANOVA) combined with a Tukey's test for post hoc analysis, with a *p*‐value < 0.05 considered statistically significant.

## 3 Results and Discussion

### 3.1 CDngs Characterization

The size and the PDI of CDngs were evaluated via DLS analysis (Figure S3) resulting in values being less than 10 nm. Similar DLS analyses have been reported in our previous work [19]. CDngs possess a stronger hydrophobic environment than the hydrophobic cavity of the corresponding CD, due to the internal space formed by the cross‐linking regions. The magnitude of this hydrophobic environment was estimated from spectral changes (Figures S4–S6) observed when various CDs and their corresponding CDngs were added to sodium 6‐(4‐*tert*‐butylaniline)‐2‐naphthalenesulfonate (BNS) aqueous solution, an environmentally responsive fluorescent probe. BNS is known to present polarity‐sensitive properties, which exhibit a short‐wavelength shift in its peak wavelength and increases fluorescence intensity in increasingly hydrophobic environments [30, 31, 32, 33, 34] The results in Figure S4 indicate that the addition of *β*‐CDngs and *γ*‐CDngs, in particular, enhances hydrophobicity. The hydrophobicity estimated from the calibration plot of wavelength shift (Figures S5 and S6) corresponds to approximately DMSO/H_2_O 93:7 for *γ*‐CDngs (Table S1) [19]. This clearly facilitates the retention of hydrophobic drugs.

### 3.2 Formulation and Quantification

In order to evaluate the feasibility of using CDngs as DDSs, we selected two different active ingredients from the classes of coumarins (OST) and stilbenoids (Combretastatin A‐4, CA‐4). OST and CA‐4 are both insoluble in water and have very similar lipophilicity with log *P* = 3.34 for OST and log *P* = 3.02 for CA‐4 (consensus log *P* as calculated with SwissADME) [35].

Regarding OST, it has shown activity toward several cancer cell lines, including leukemia (IC_50_ = 38.1–61.0 µM) [36], human colon adenocarcinoma (IC_50_ = 122.5 μM) [36], cervical carcinoma (IC_50_ = 77.8 µM) [37], and hepatocellular carcinoma (137.0–161.9 µM) [38]. Little evidence was reported for the use of OST in combination with CDs, suggesting the formation of inclusion complexes [39, 40].

On the other hand, CA‐4 has shown broad in vitro antiproliferative effects across numerous human tumor cell lines, including those with multidrug resistance [41]. To the best of our knowledge, there are no reports on CA‐4 in combination with CDs; a previous study reports the use of graphene‐oxide nanoparticles conjugated with β‐CD showing the interesting ratio of IC_50_^CA−4 pure^/IC_50_^CA−4 DDS^ = 2.37/0.44 nM (MG‐63 cell lines) [42].

**FIGURE 2:**
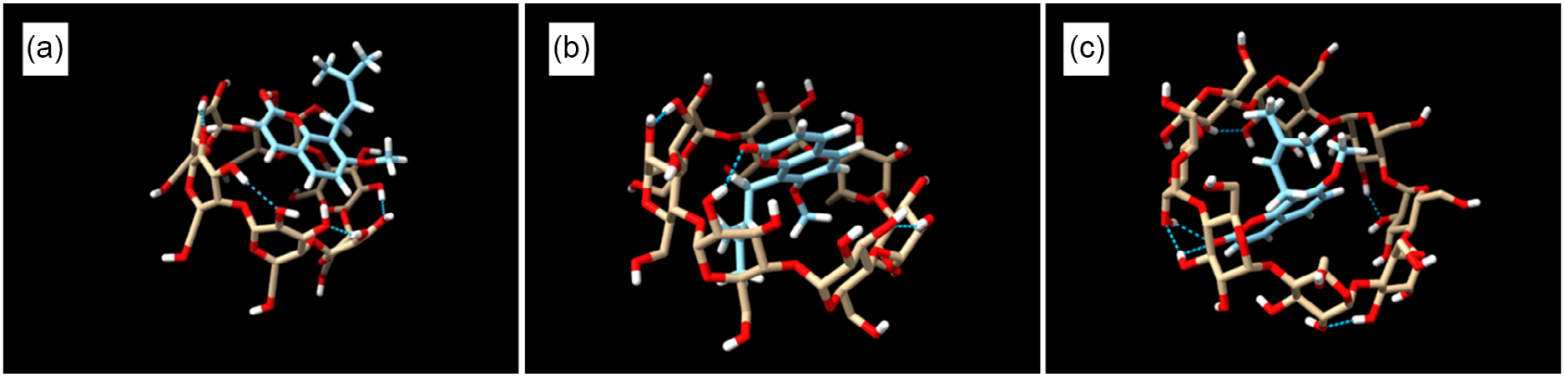
Best docking mode [distance from RMSD (l.b.) = 0, Autodock Vina] for OST/α‐CD (a), OST/β‐CD (b), and OST/γ‐CD (c).

Our investigation started with 1:1 OST and CDs mixtures, prepared by continuous stirring (8 h) of a saturated solution of the drug at a fixed theoretical concentration (5 mM), with and without the CDs. Since the nanogels were prepared from α‐CD, β‐CD, and γ‐CD, the same CD were selected in order to preliminarily test the affinity of OST with native CDs. In Figure S7, it is reported the comparison screening with CDs as shown by ^1^H NMR spectra acquired under the same conditions. By quantification of the integrated areas of aromatic signals of OST (H_2_, H_3_, H_4_, and H_1_) it is possible to observe that β‐CD produced the greatest solubility enhancement (∼9.5× compared to pure OST, as calculated by the NMR signals integrated area). Complexation shifts were in accordance with the solubility enhancement trend, attributing the highest interaction to OST/β‐CD, but still with small variation with respect to pure OST (Table S2). This behavior was confirmed and better rationalized by docking analysis (Autodock Vina). As reported in Figure 2, OST tends to have superficial interaction with α‐CD, better‐fitting inclusion with β‐CD, and a looser inclusion with γ‐CD (Tables S3–S5). The association constant found in the literature for β‐CD is *K*_a_ = 1.23 × 10^2^ M^−1^ [40], whereas the calculated constant based on the Δ*G*_bind_ from our docking analysis is *K*_a_^calc^ = 3.23 ± 0.43 x 10^3^ M^−1^.

**FIGURE 3:**
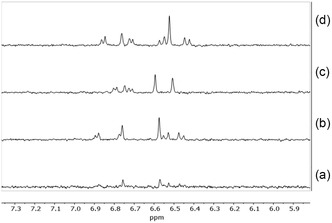
^1^H NMR spectra (500 MHz, D_2_O, 25 °C) of (a) CA‐4; (b) CA‐4/α‐CD; (c) CA‐4/β‐CD; and (d) CA‐4/γ‐CD.

On the basis of these results, β‐CD was identified as the best host for our purpose. The increase in solubility was correlated with β‐CD concentration (Figure S8) and the mixture was confirmed to be stable after 1 month (Figure S9). For comparing the mixtures OST/β‐CD and OST/β‐CDngs a simple formulation procedure was conducted (see sample preparation section). Unfortunately, the ^1^H NMR spectra show no significant improvements in OST solubility with the β‐CDngs with respect to β‐CD, even with the addition of 10% DMSO to predissolve OST (Figure S8, EE% ≈ 8%). Hence, it is possible to confirm that the interactions established by OST and β‐CDngs are similar to those between OST and β‐CD, without further contribution due to the crosslinked structure. Since EE% < 10% was considered unsatisfactory, we then moved to evaluate the use of CA‐4. Unlike OST, α‐CD and γ‐CD produce the best solubility enhancement respect to pure CA‐4 (Figure 3a–d), with γ‐CD slightly better than α‐CD (3.6× vs. 4.7×, respectively, as calculated with respect to the integrated area of pure CA‐4).

Complexation shift for CA‐4 showed interesting results (Table 1), indicating that ring A is involved in the inclusion for all the CDs. In addition to that, relevant shielding was detected also for ring B only in the case of γ‐CD.

**TABLE 1:** ^1^H NMR (500 MHz, D_2_O, 25 °C) chemical shift of pure CA‐4 and complexation shift of CA‐4 in mixture with α‐CD, β‐CD, and γ‐CD (1:5).

		*δ*, ppm	Δ*δ*, ppm	Δ*δ*, ppm	Δ*δ*, ppm
		CA‐4	CA‐4/α‐CD	CA‐4/β‐CD	CA‐4/γ‐CD
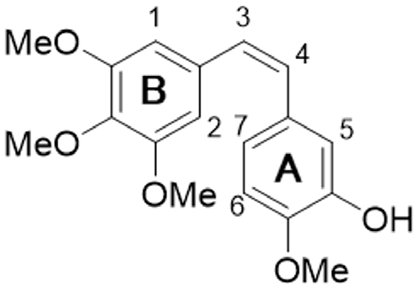	H_1/2_	6.57	0.00	0.02	−0.05
	H_3_	6.51	0.04	0.00	0.06
	H_4_	6.41	0.07	0.11	0.04
	H_5_	6.64	0.11	0.10	0.12
	H_6_	6.61	0.17	0.12	0.12
	H_7_	6.81	0.08	−0.01	0.05

**FIGURE 4:**
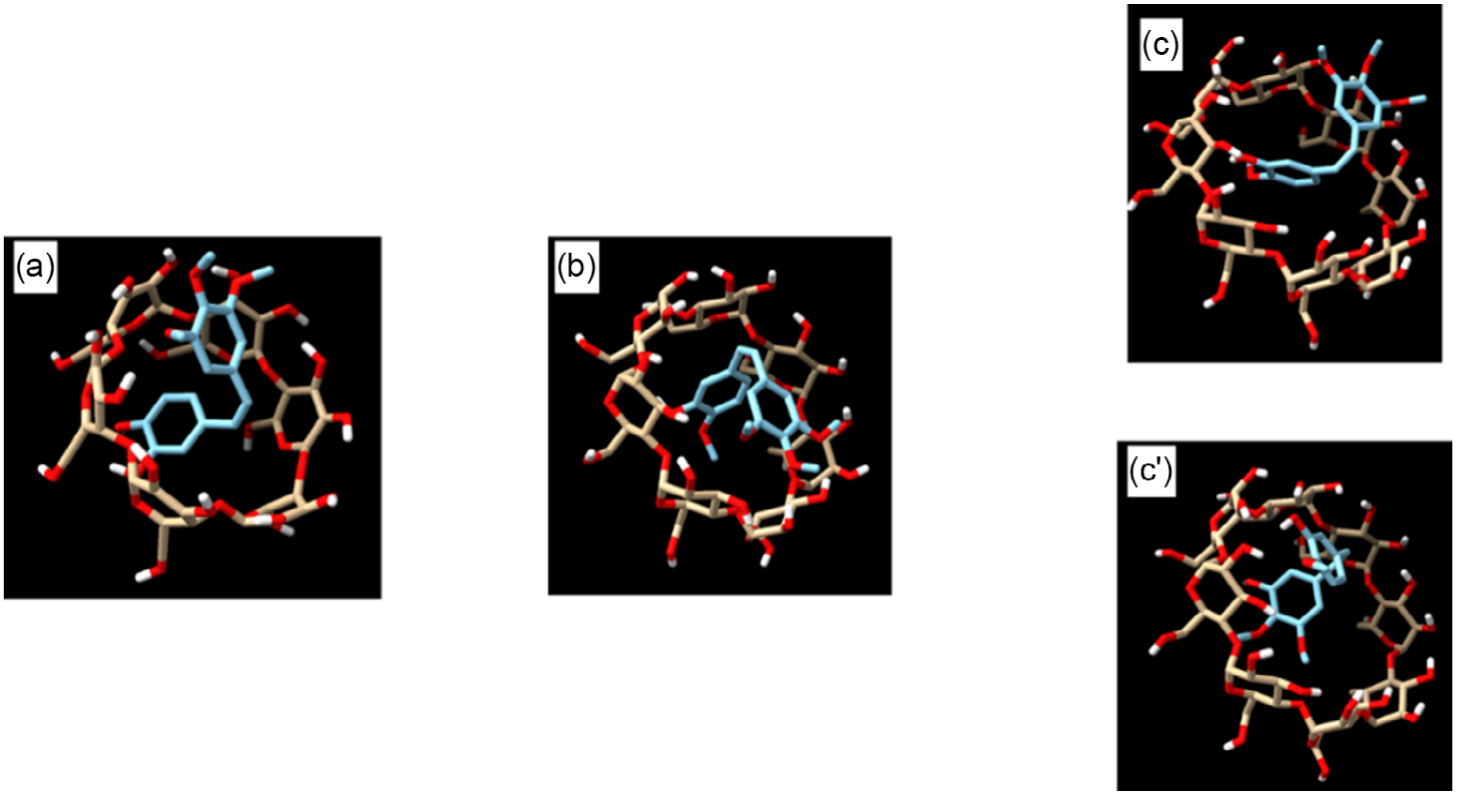
Best docking mode [Distance from RMSD (l.b.) = 0, Autodock Vina] for CA‐4/α‐CD (a), CA‐4/β‐CD (b) and CA‐4/γ‐CD (c). Additional binding mode (rank = 3) for CA‐4/γ‐CD (c’).

Also, in the case of CA‐4/CD system, a docking analysis was conducted (Figure 4). In contrast with OST, α‐CD is capable of successfully including ring A, as is β‐CD. In the case of γ‐CD, different binding poses with relatively high score were found where CA‐4 was included also from ring B (Figure 4c’, Tables S6–S8).

The same formulation used with OST was carried out for CA‐4. EE% and solubility enhancement reflected the findings obtained, supporting α‐CD and γ‐CD as the best candidates, with slight preference for the latter (Table S9). Overall, considering the more extensive interaction, the higher EE% and solubility enhancement, γ‐CD can be considered the best host. Hence, we therefore prepared a formulation using the corresponding γ‐CDngs. This led to a CA‐4/γ‐CDngs loaded nanogel with EE% considered satisfactory (EE = 22.4%) to carry out further cell line evaluation.

It is worth noting that an EE% of 22.4% (and DL ≈ 1%), while appearing moderate compared to bulk hydrogels or larger microparticles, represents a significant achievement given the formulation strategy strictly required for these dimensionally constrained architectures (<10 nm, Figure S3). In this study, the active principle was deliberately loaded after the nanogel synthesis (postloading) to preserve the delicate, recently optimized morphology of the empty carriers. In such preformed nanoscale networks, the EGDE crosslinks sterically compete with the payload for the limited internal free volume. Achieving this loading capacity via physical diffusion, without inducing drug precipitation or carrier aggregation, confirms the structural integrity of the γ‐CDngs system and represents an optimal thermodynamic balance for a postloading procedure.

### 3.3 Cytotoxicity Assessment

**FIGURE 5:**
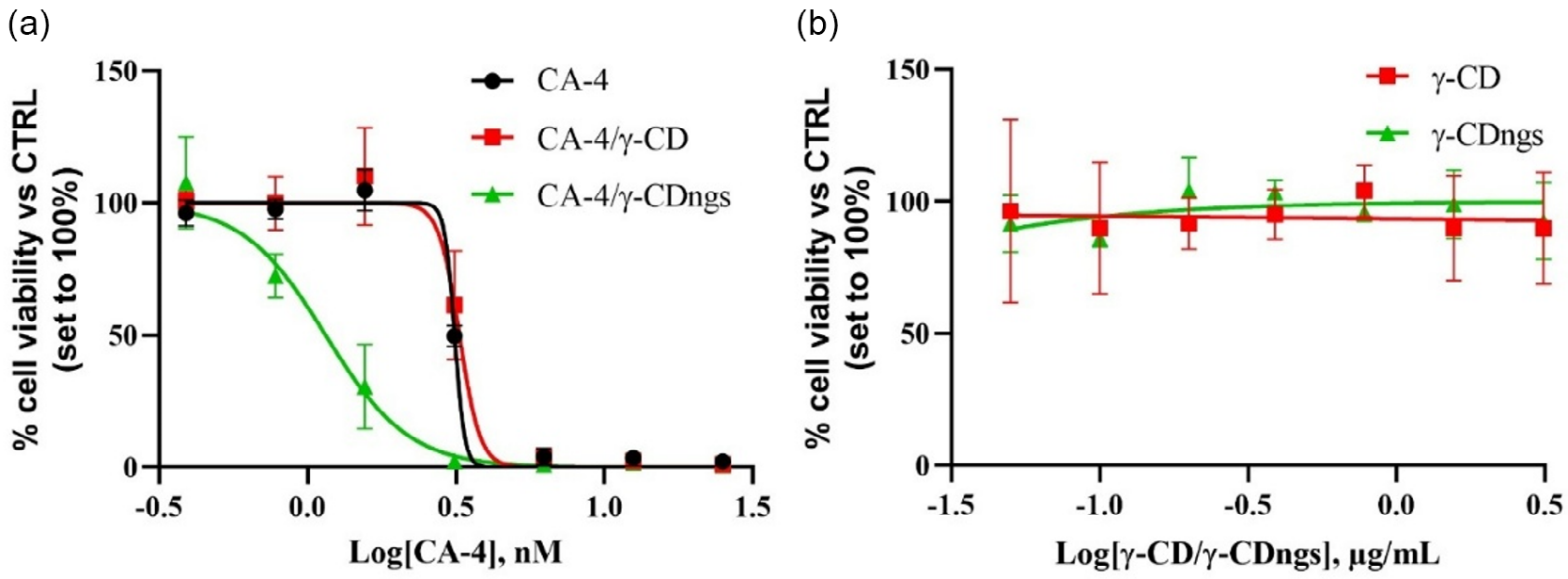
A2780 human ovarian cancer cell line viability after exposure for 72 h to free CA‐4 and the CA‐4‐loaded (a) and unloaded (b) γ‐CD(ngs) systems at various concentrations. Data are reported as mean ± standard deviation (*n* = 3).

To evaluate the potential chemotoxicity of the developed DDS, the A2780 cell line was employed (Figure 5a). Indeed, previous studies have demonstrated a strong concentration‐dependent cytotoxic activity of CA‐4 against this human ovarian cancer cell line. The IC_50_ of free CA‐4 was 3.16 ± 0.08 nM (Table 2) in agreement with those previously found by Schmidt C. et al. (IC_50_ = 2.37 nM) [43], Faouzi et al. (IC_50_ = 2.5 nM) [44], and Guazzelli et al. (IC_50_ = 2.0 nM) [45].

**TABLE 2:** IC_50_ values of free CA‐4, as well as CA‐4‐loaded and unloaded γ‐CD or γ‐CDngs systems against the A2780 cell line.

IC_50_ 72 h – A2780 cell line				
nM			µg/mL	
CA‐4	CA‐4/γ‐CD	CA‐4/γ‐CDngs	γ‐CD	γ‐CDngs
3.16 ± 0.08	3.4 ± 0.5	1.2 ± 0.2***	>3.13	>3.13

*Note*: Data are reported as mean ± standard deviation (*n* = 3).

***Value significantly different from the others (*p* < 0.001).

In the case of the CA‐4/γ‐CD IC_50_ value (3.4 ± 0.5 nM) statistically comparable to that of free CA‐4 was obtained, indicating that the drug encapsulation did not alter its cytotoxicity. However, a significant decrease (*p* < 0.001) of IC_50_ to 1.2 ± 0.2 nM was observed in the case of CA‐4/γ‐CDngs. This enhanced chemotoxicity may be attributed to several factors, including improved cellular uptake facilitated by the dimensions of CDngs in comparison to individual CD. The CDngs formation is known to enhance drug bioavailability by providing protection against deleterious environmental factors, such as pH fluctuations, temperature variations, and light exposure [46].

Furthermore, the significant reduction in IC_50_ observed at 72 h (1.2 nM vs. 3.16 nM for the free drug) provides functional evidence that the payload remains bioavailable and is efficiently released from the nanogel network following cellular internalization. Owing to their hydrophilic character, CDngs are expected to be internalized predominantly via endocytic pathways [47], delivering the encapsulated CA‐4 to the intracellular, and presumably endolysosomal, environment [48], where the host–guest equilibrium can shift toward payload release. This functional readout, together with the protective effect against environmental degradation described above, offers an important complement to standard extracellular dialysis‐based release assays, whose interpretation for ultrasmall, highly hydrated nanogels carrying poorly water‐soluble payloads is intrinsically limited: the slow passive diffusion of a highly lipophilic drug such as CA‐4 across an aqueous‐filled dialysis membrane can itself become rate‐limiting, generating release profiles that reflect membrane‐diffusion artifacts rather than the true intracellular release dynamics. The full cytocompatibility of the unloaded systems (Figure 5b) indicates that both γ‐CD and γ‐CDngs did not contribute to the resulting cytotoxic effects.

## 4 Conclusion

This work presents the first attempt to use ultrasmall CDngs as DDS. We confirmed that this type of CDngs cross‐linked with EGDE are not intrinsically cytotoxic toward the cell line selected. The choice of the active ingredient must rely on its chemical structure involving both CD inclusion and superficial/interstitial interactions with the nanogel supramolecular framework, i.e., the interactions within the nanogel network are not a simple extension of CD host–guest chemistry but instead reflect more complex affinity patterns. In the case of OST, the nanostructure has not overcome the limited interaction with the β‐CD in our experimental setup. On the other hand, CA‐4 proved to be the ideal candidate for γ‐CD, and even more with γ‐CDngs, due to dual possible inclusion phenomena involving the more hydrophobic (methoxyphenol ring A) and less hydrophobic (trimethoxy‐aromatic ring B) moieties. Despite γ‐CD showing theoretically lower affinity for the guest, in this case, its wider cavity represents a distinct advantage, also in terms of EE%. Even with moderate drug‐loading values, the formulations maintained biological efficacy, validating the suitability of this platform with an IC50 toward the A2780 cell line of 3.4 nM in the case of γ‐CD and 1.2 nM in the case of γ‐CDngs. This simple formulation strategy therefore provides a viable route for delivering poorly soluble molecules. Future studies will aim to maximize the EE% by performing the crosslinking step directly in the presence of the active ingredient. Such an in situ loading approach will require a dedicated, extensive synthetic optimization to ensure that the chosen drug strictly meets three criteria: (1) it must be chemically inert toward the highly reactive epoxide groups of the EGDE crosslinker, (2) it must be fully compatible with the complex biphasic surfactant‐assisted environment, and (3) its physical encapsulation during the crosslinking phase must not alter the delicate interfacial thermodynamics required to maintain the highly desirable ultrasmall dimensions of the carriers. Further biological evaluations, including the covalent tagging of the nanogel network to perform comprehensive confocal fluorescence microscopy, are currently being planned. This will elucidate the active intracellular uptake pathways and detailed endosomal trafficking without relying on noncovalent dyes that might suffer from premature leakage. Overall, these findings open the way for CDngs as a promising and adaptable system for drug delivery.

## Funding

This work was supported by the European Union – Next Generation EU through the Italian Ministry of University and Research under PNRR ‐ M4C2‐I1.3 Project PE_00000019 “HEAL ITALIA” (CUP I53C22001440006).

## Conflicts of Interest

The authors declare no conflicts of interest.

## Supporting information

Supplementary Material

## Data Availability

Research data are not shared.
