# Supplementary material for: Ultrasmall Cyclodextrin‐Based Nanogels as Drug Delivery Systems
Source: ChemistryOpen. 2026 Aug 4;15(8):e70274. doi: 10.1002/open.70274 (PMC13437435; doi:10.1002/open.70274)
Supplement: Supplementary file 1 [file OPEN-15-e70274-s001.pdf]

Supporting Information

Ultrasmall cyclodextrin-based nanogels as drug delivery systems

Andrea Cesari,*[a] Simona Braccini,[a] Maria Antonietta Casulli,[b] Kento Ishigaki,[c] Takeshi Hashimoto,[c] Takashi Hayashita,[c] Dario Puppi,[a] Fabio Bellina[a]

[a] Department of Chemistry and industrial Chemistry, University of Pisa, Via G. Moruzzi, Pisa, 56124

[b] Micro and Nano Systems (MNS), Leuven (Arenberg), Arenberg Castle Park 10, 3001 Leuven

[c] Graduate School of Science and Technology, Department of Materials and Life Sciences, Faculty of Science and Technology, Sophia University, 7-1 Kioi-cho, Chiyoda-ku, Tokyo 102-8554, Japan

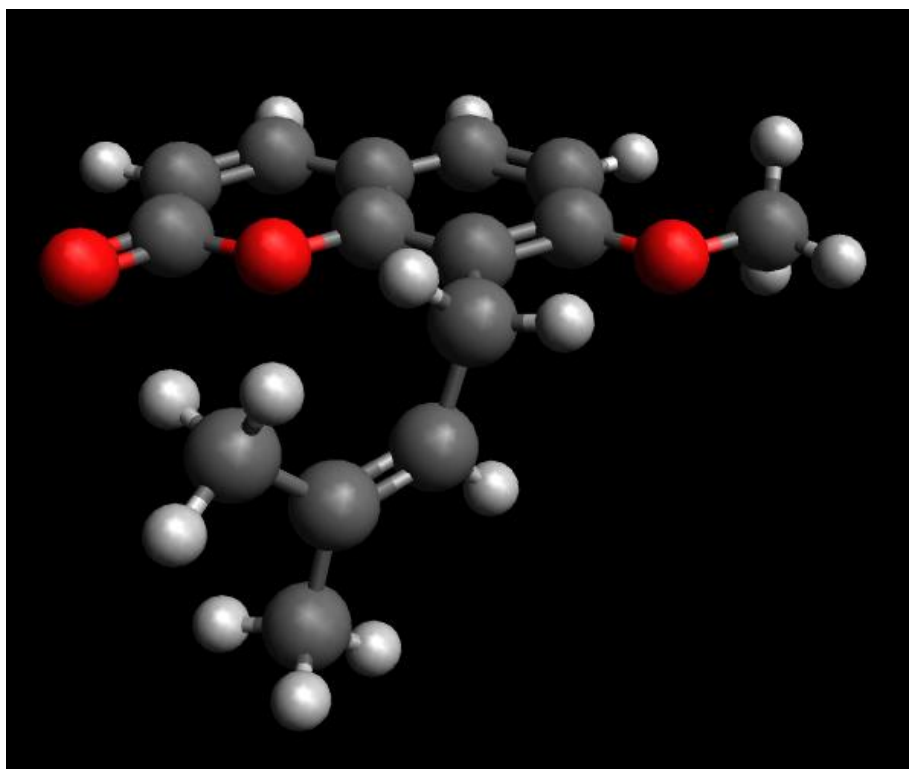

Figure S1. ORCA Global Optimizer Algorithm (GOAT) minimum conformation of Osthole (Ost).

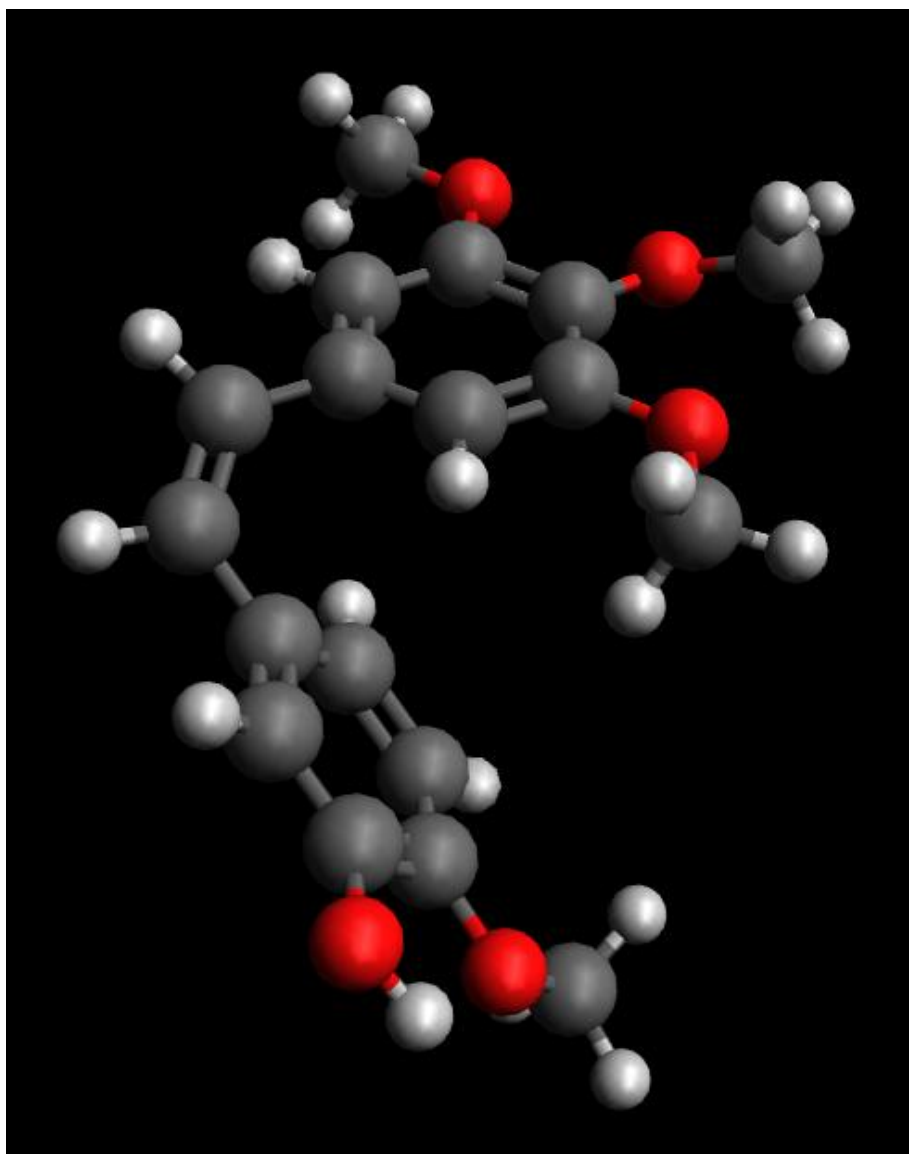

Figure S2. ORCA Global Optimizer Algorithm (GOAT) minimum conformation of Combretastatin A-4 (CA-4).

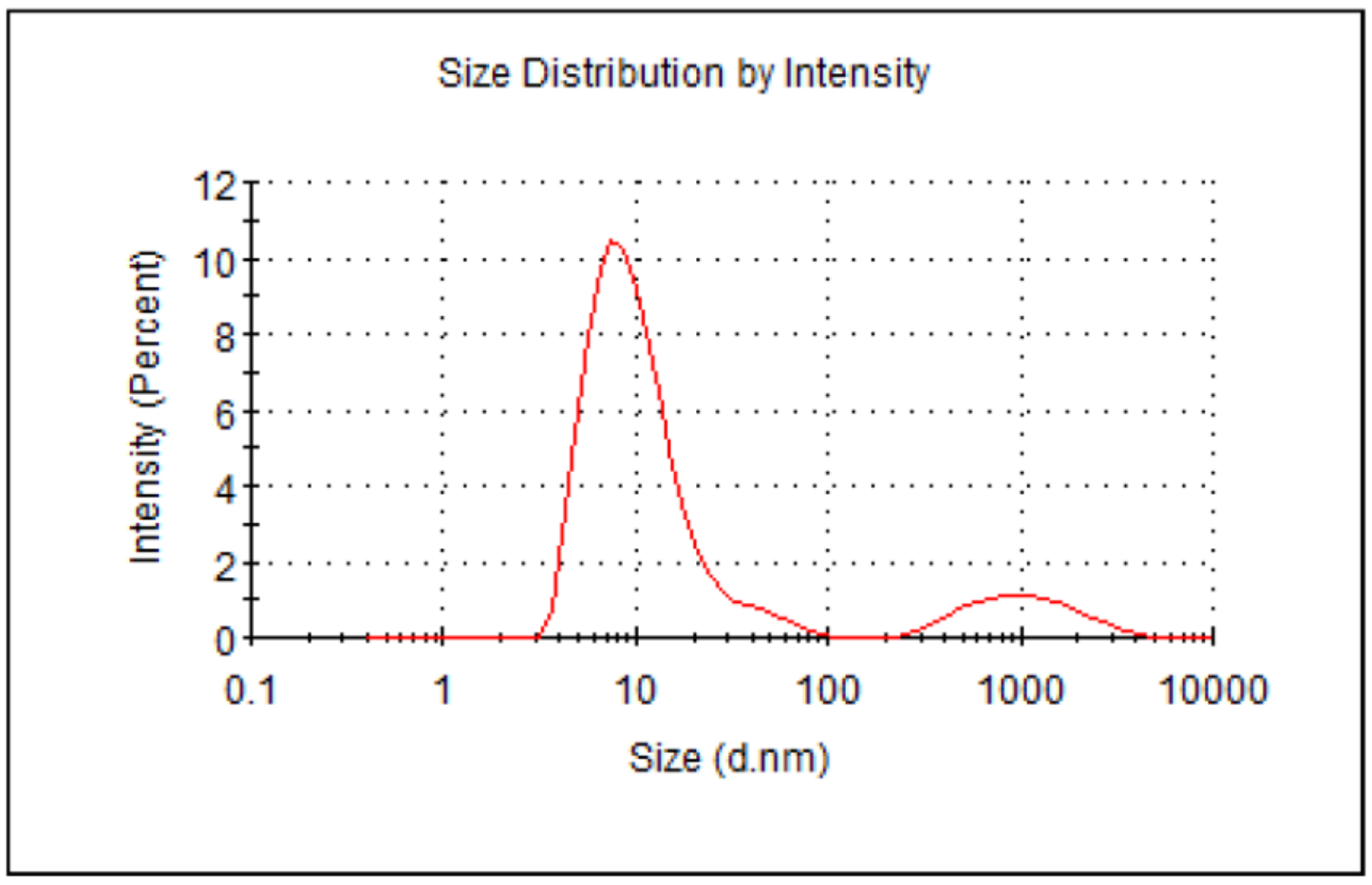

1
Diameter = 4.865
PdI = 0.329

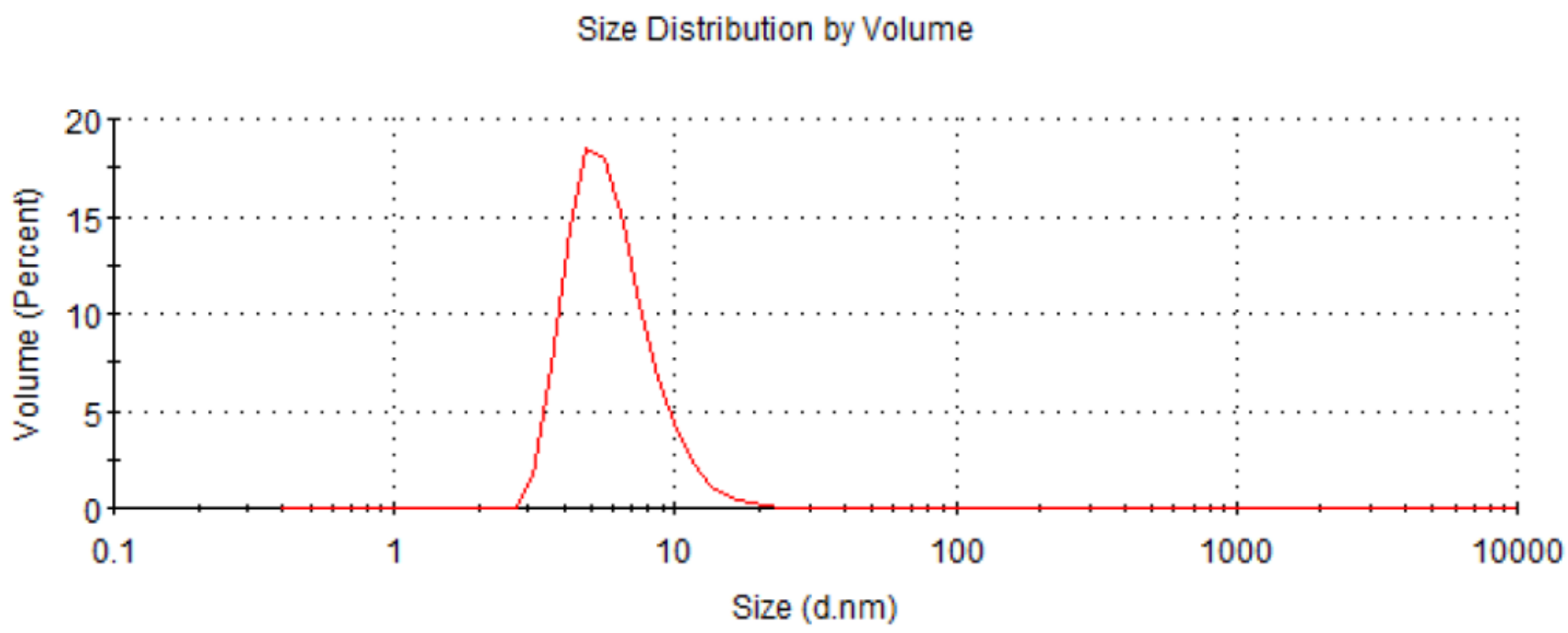

1
Diameter = 4.865
PdI = 0.329

Figure S3. DLS data (nanogel diameter and polydispersity index (PDI) of γ-CyDngs.

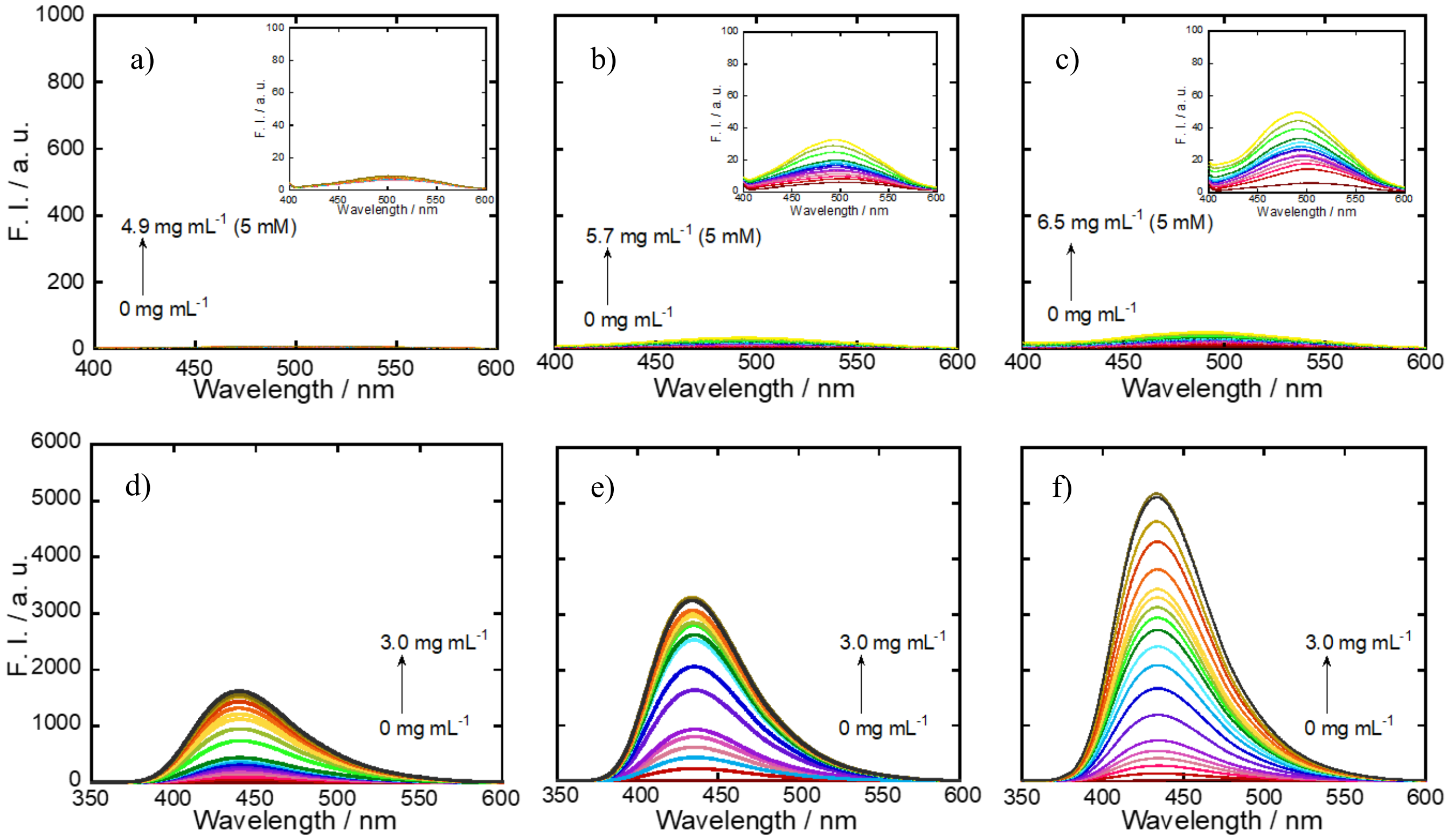

Figure S4. Fluorescence spectral changes of BNS/CD or CDngs as CD's concentration for a) α-CD; b) *β*-CD; c) *γ*-CD; d) α-CDngs; e) *β*-CDngs; f) *γ*-CDngs. [BNS] = 10 uM, [CD] = 0.0 - 5.0 mM, [CDngs] = 0.0-3.0 mg/mL, pH 7.4 adjusted by HEPES-NaOH buffer in 1% DMSO-99% water, at 25℃. λ_{ex} = 320 nm.

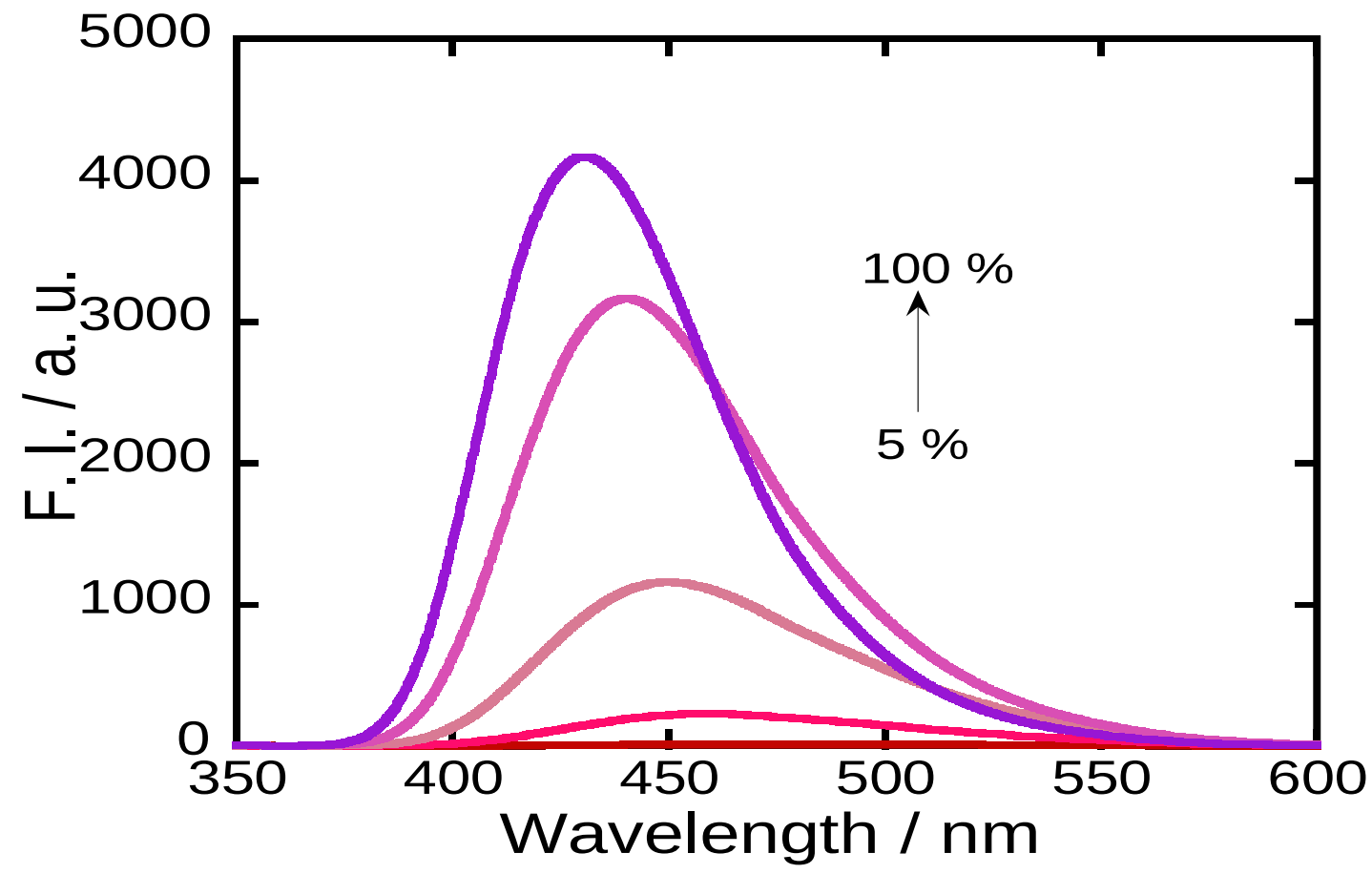

Figure S5. Fluorescence spectra of BNS in DMSO/water (v/v, %), at 25℃ (λ_{ex} = 320 nm). [BNS] = 1.0 × 10^{-5} M, DMSO = 5 - 100 %

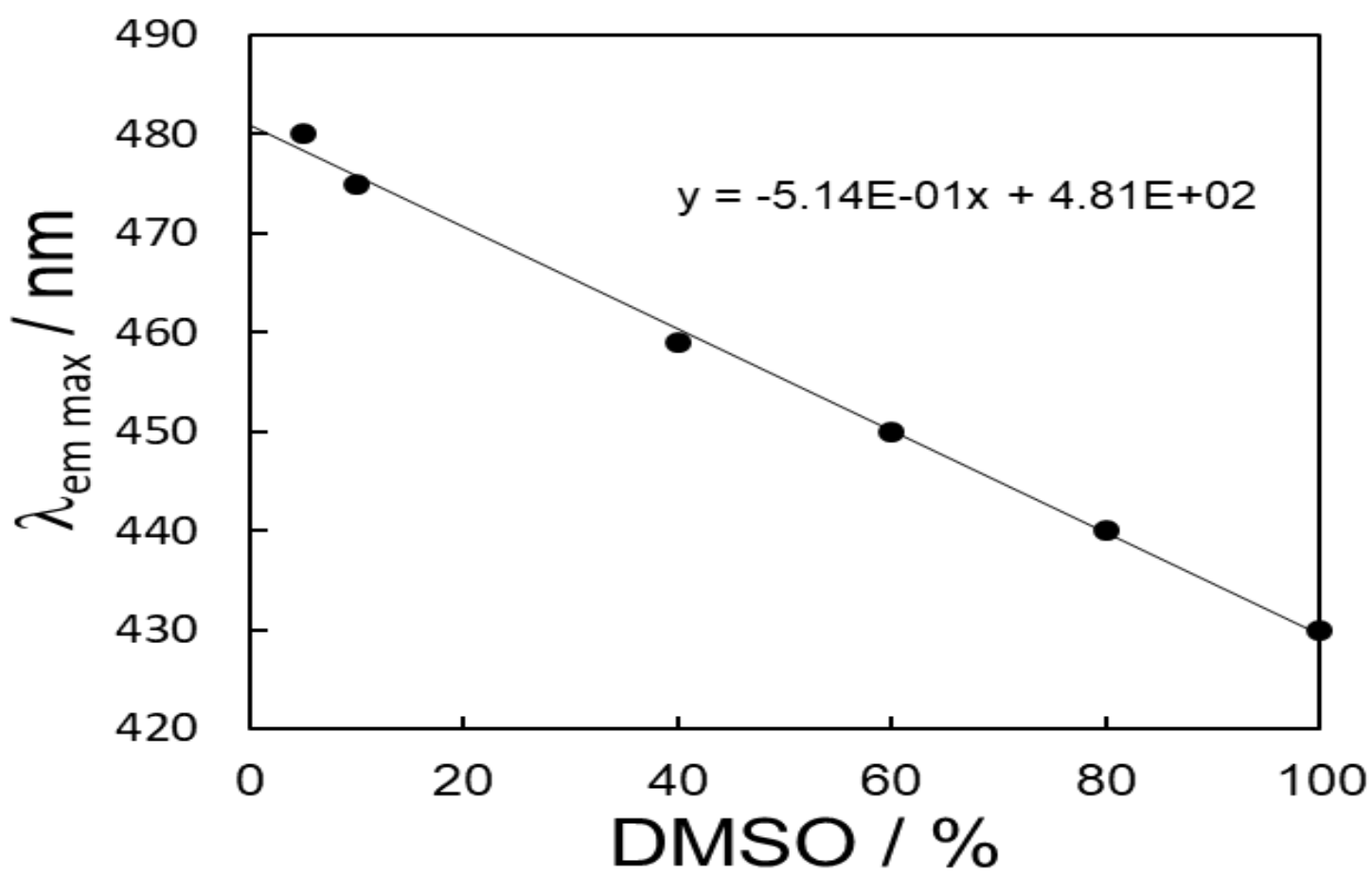

Figure S6. A plot of fluorescence maximum wavelength ($\lambda_{ex\text{-}max}$) vs. DMSO mixing ratio (v/v, %).

Table S1. Fluorescence max wavelength ($\lambda_{em\text{-}max}$) of BNS/CD or CDngs complexes.

Complex	$\lambda_{em\text{-}max}$ / nm	DMSO / % #
BNS/*α*-CD	455	50
BNS/*β*-CD	440	80
BNS/*γ*-CD	454	53
BNS/*α*-CDngs	441	78
BNS/*β*-CDngs	434	91
BNS/*γ*-CDngs	433	93

DMSO % is estimated from Takeuchi, S.; Cesari, A.; Soma, S.; Suzuki, Y.; Casulli, M. A.; Sato, K.; Mancin, F.; Hashimoto, T.; Hayashita, T. Preparation of Ultrasmall Cyclodextrin Nanogels by an Inverse Emulsion Method Using a Cationic Surfactant. Chem. Commun. 2023, 59 (27), 4071–4074. https://doi.org/10.1039/d3cc00523b.

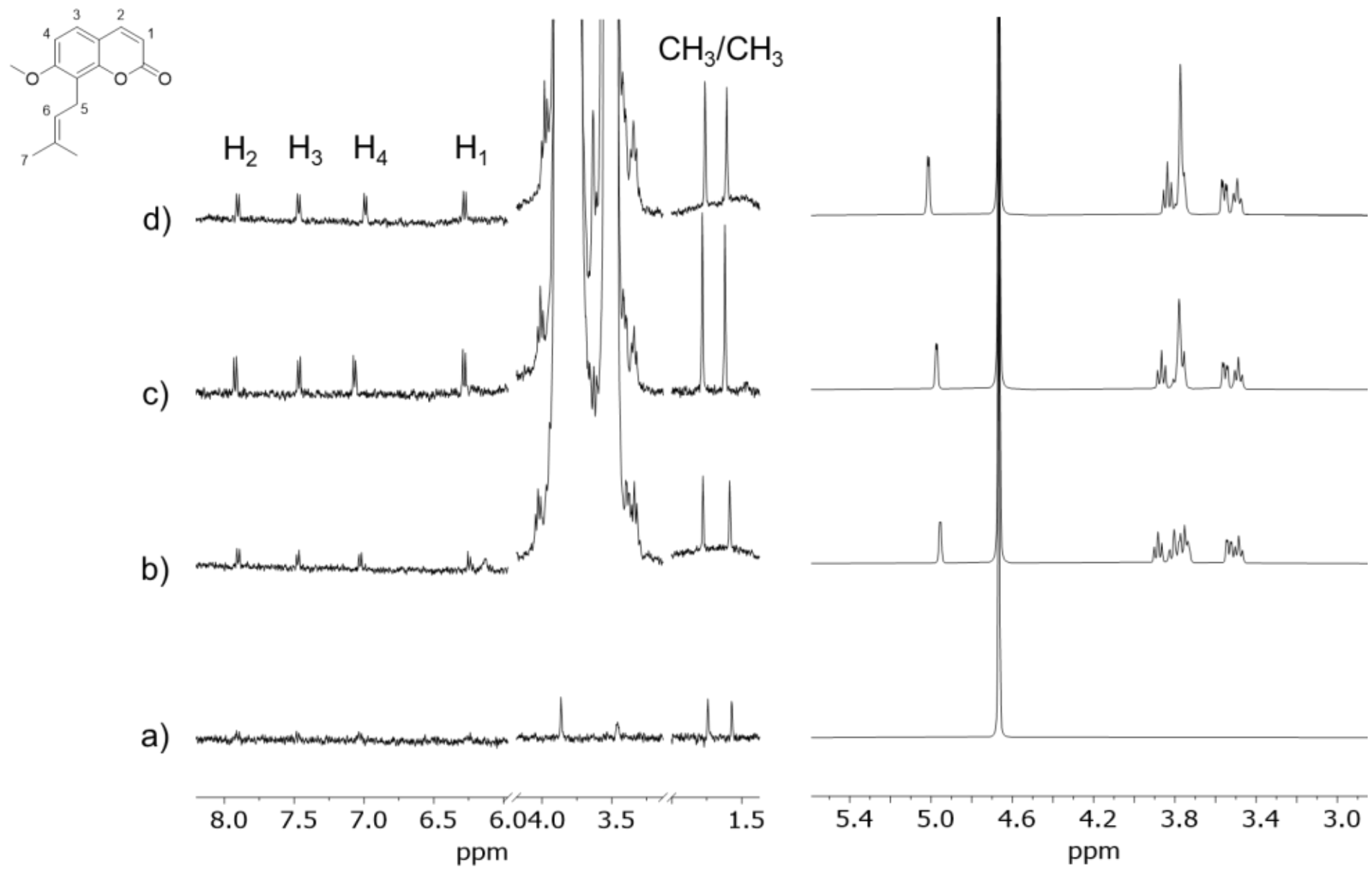

Figure S7. ^{1}H NMR (500 MHz, 25 °C, D_2O) of a) OST; b) OST/α-CD; c) OST/β-CD; d) OST/γ-CD (1:1 mixture, 5 mM).

Table S2. ^{1}H NMR (500 MHz, D_2O, 25 °C) chemical shift of pure CA-4 and complexation shift of CA-4 in mixture with α-CD, β-CD, and γ-CD (1:1).

**Proton**	**δ (ppm)**	**Δδ (ppm) α-CD**	**Δδ (ppm) β-CD**	**Δδ (ppm) γ-CD**
H_1	6.25	0.00	0.03	0.03
H_2	7.90	0.00	0.02	0.00
H_3	7.47	0.00	-0.01	-0.01
H_4	7.02	0.00	0.05	-0.03
H_7/H_8	1.74/1.57	0.04/0.02	0.04/0.05	0.02/0.04

Table S3. OST/α-CD; predicted binding modes generated by AutoDock Vina, with corresponding binding free energies (ΔG) and RMSD values relative to the top-ranked mode.

Mode	ΔG (kcal/mol)	Distance from RMSD (l.b.)	Best mode RMSD (l.b.)
1	-4.263	0	0
2	-4.259	2.847	5.576
3	-4.224	2.591	4.476
4	-4.178	2.818	4.715
5	-4.131	2.498	5.219
6	-4.119	2.445	4.309
7	-4.071	2.625	5.305
8	-4.058	2.648	5.405
9	-4.046	3.293	5.549
10	-4.044	2.506	5.178
11	-3.986	2.024	4.553
12	-3.986	2.737	4.436
13	-3.979	2.419	3.94
14	-3.944	3.171	4.661
15	-3.928	2.574	3.742
16	-3.883	1.842	3.837
17	-3.875	1.869	5.805
18	-3.871	7.553	9.021
19	-3.869	2.222	4.046
20	-3.853	2.175	4.404

Table S4. OST/β-CD; predicted binding modes generated by AutoDock Vina, with corresponding binding free energies (ΔG) and RMSD values relative to the top-ranked mode.

Mode	ΔG (kcal/mol)	Distance from RMSD (l.b.)	Best mode RMSD (l.b.)
1	-4.898	0	0
2	-4.802	2.252	3.791
3	-4.801	2.355	3.027
4	-4.757	2.088	3.119
5	-4.68	2.125	3.454
6	-4.633	2.377	3.177
7	-4.625	1.744	1.966
8	-4.473	2.521	4.198
9	-4.352	2.412	6.036
10	-4.343	2.985	4.381
11	-4.328	2.269	5.439
12	-4.296	2.287	6.682
13	-4.283	1.74	6.072
14	-4.267	2.07	3.292
15	-4.255	1.992	5.272
16	-4.244	3.497	5.035
17	-4.223	2.379	3.459

18	-4.221	2.031	5.379
19	-4.185	2.318	5.986
20	-4.172	3.198	4.357

Table S5. OST/γ-CD; predicted binding modes generated by AutoDock Vina, with corresponding binding free energies (ΔG) and RMSD values relative to the top-ranked mode.

Mode	ΔG (kcal/mol)	Distance from RMSD (l.b.)	Best mode RMSD (l.b.)
1	-4.508	0	0
2	-4.506	2.251	2.825
3	-4.504	2.184	4.03
4	-4.502	2.592	4.066
5	-4.502	2.268	4.63
6	-4.501	2.526	4.381
7	-4.5	2.269	2.852
8	-4.482	1.702	2.031
9	-4.467	2.537	5.353
10	-4.454	2.624	6.093
11	-4.452	2.157	4.963
12	-4.445	2.409	3.66
13	-4.442	2.644	4.733
14	-4.436	2.133	5.76
15	-4.412	2.44	4.28
16	-4.35	2.332	5.233
17	-4.342	2.238	5.101
18	-4.338	2.328	5.927
19	-4.333	2.801	4
20	-4.321	1.893	6.006

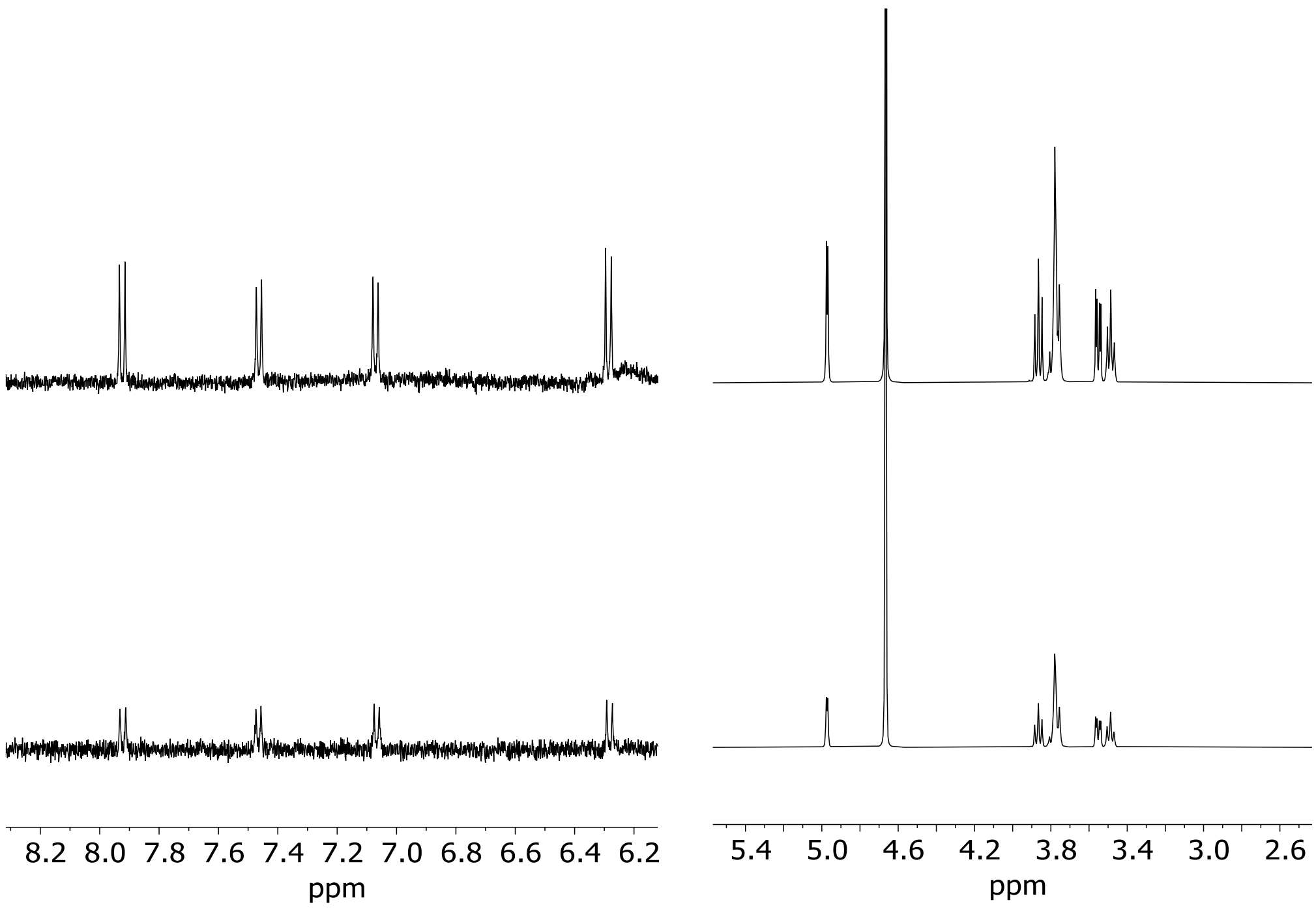

Figure S8. Comparison of ^1H NMR (500 MHz, D_2O, 25 °C) for OST/CyD mixture with 1 eq (down) and 3 eq (up) of β-CyD.

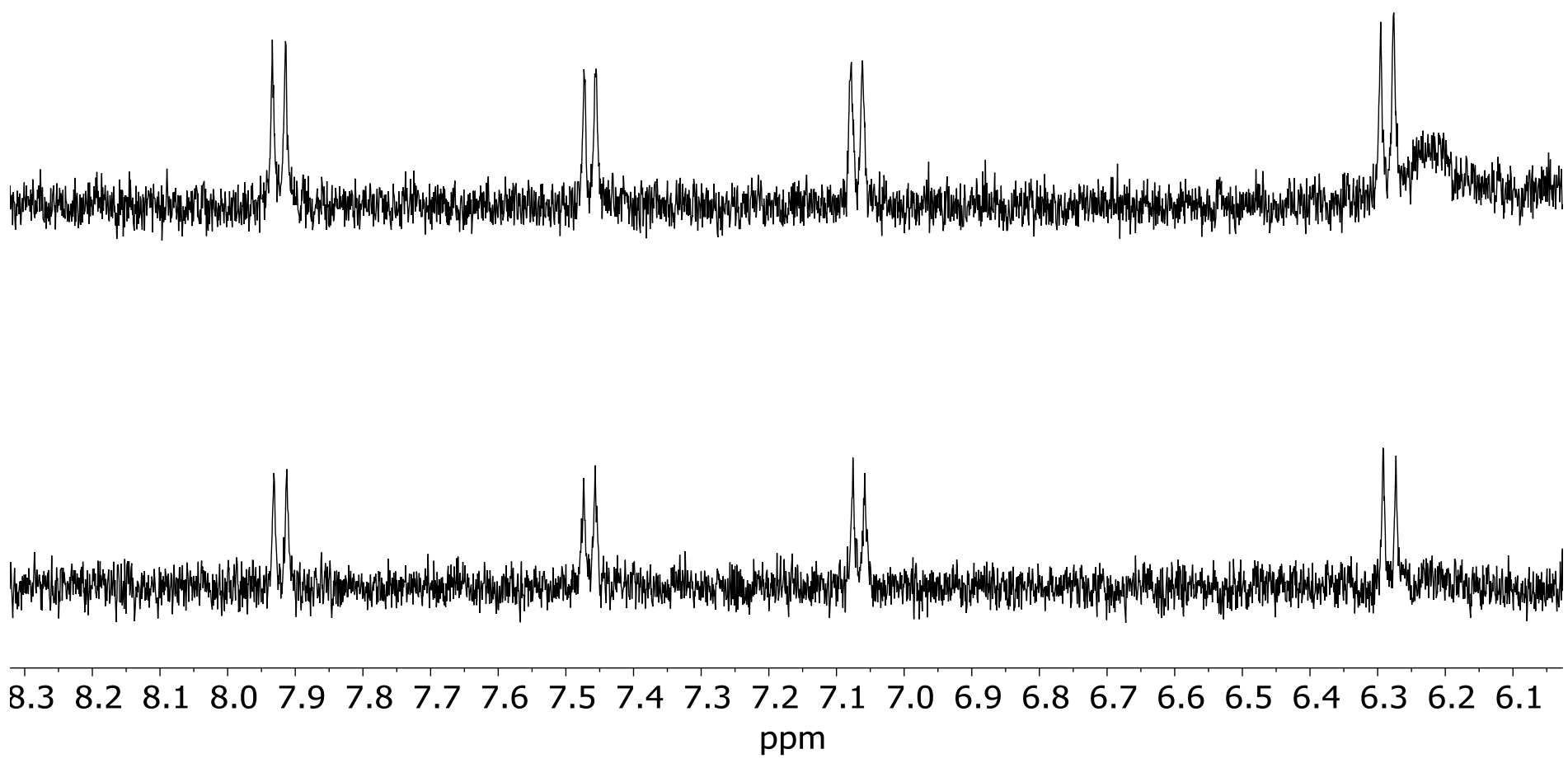

Figure S9. Comparison of ^1H NMR (500 MHz, D_2O, 25 °C) for OST/β-CD 1:1 mixture (5 mM) at t_0 (down) and after 1 month (up).

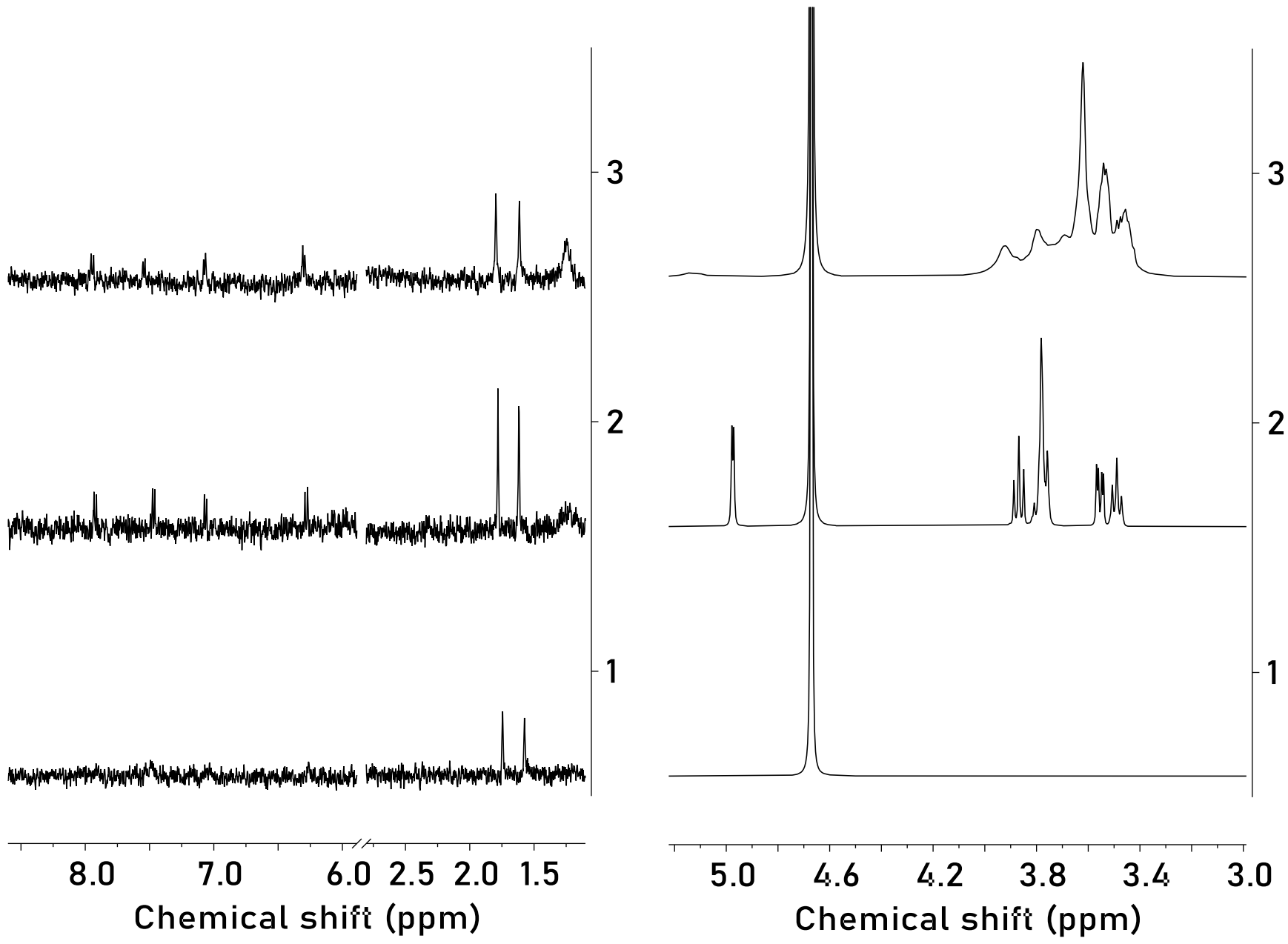

Figure S10. Comparison of ^{1}H NMR (500 MHz, D_2O+10% DMSO-d_6, 25 °C) for OST (1), OST/β-CD (2), and OST/β-CDngs (3).

Table S6. CA-4/α-CD; predicted binding modes generated by AutoDock Vina, with corresponding binding free energies (ΔG) and RMSD values relative to the top-ranked mode.

Mode	ΔG (kcal/mol)	Distance from RMSD (l.b.)	Best mode RMSD (l.b.)
1	-5.996	0	0
2	-5.969	0.3266	2.538
3	-5.64	3.235	4.366
4	-5.546	1.681	3.027
5	-5.516	2.783	4.508
6	-5.499	2.965	4.353
7	-5.28	2.206	4.042
8	-5.23	2.576	4.283
9	-5.169	2.599	3.889
10	-5.151	3.257	5.87
11	-5.069	3.265	5.272
12	-5.044	3.578	6.664
13	-5.039	3.502	5.737
14	-4.99	3.579	6.297
15	-4.957	2.288	3.886
16	-4.944	3.54	6.122
17	-4.943	3.657	8.65
18	-4.9	3.29	4.318
19	-4.845	3.389	6.641
20	-4.823	3.491	5.306

Table S7. β-CD/OST; predicted binding modes generated by AutoDock Vina, with corresponding binding free energies (ΔG) and RMSD values relative to the top-ranked mode.

Mode	ΔG (kcal/mol)	Distance from RMSD (l.b.)	Best mode RMSD (l.b.)
1	-5.757	0	0
2	-5.652	2.377	4.068
3	-5.635	0.156	2.664
4	-5.611	2.504	4.07
5	-5.609	3.034	4.331
6	-5.595	2.318	3.782
7	-5.586	1.297	2.372
8	-5.583	3.012	5.088
9	-5.578	1.338	3.094
10	-5.564	3.05	4.513
11	-5.564	2.758	4.198
12	-5.558	2.805	4.028
13	-5.554	1.667	3.38
14	-5.55	2.993	4.973
15	-5.545	1.718	2.868
16	-5.505	3.066	4.531
17	-5.484	2.674	4.313
18	-5.345	3.185	5.355
19	-5.267	3.272	5.406
20	-5.181	1.769	3.719

Table S8. γ-CD/OST; predicted binding modes generated by AutoDock Vina, with corresponding binding free energies (ΔG) and RMSD values relative to the top-ranked mode.

Mode	ΔG (kcal/mol)	Distance from RMSD (l.b.)	Best mode RMSD (l.b.)
1	-5.236	0	0
2	-5.156	3.091	4.659
3	-5.143	3.344	6.746
4	-5.139	3.939	6.511
5	-5.113	3.889	7.542
6	-5.113	3.933	7.359
7	-5.109	3.048	4.634
8	-5.104	2.688	7.233
9	-5.089	2.511	6.58
10	-5.085	1.724	7.006
11	-5.062	2.368	7.049
12	-5.06	2.713	7.016
13	-5.058	3.847	5.739
14	-5.048	4.149	7.199
15	-5.025	4.138	7.583
16	-5.013	1.855	6.626
17	-4.994	3.519	7.355
18	-4.988	2.787	7.095
19	-4.977	3.957	5.807
20	-4.976	2.214	3.184

Table S9. Encapsulation efficiency (EE%) for CA-4 mixtures as quantified by NMR spectroscopy.

Sample	EE%
CA-4/α-CD	14.3
CA-4/β-CD	4.4
CA-4/γ-CD	19.0
CA-4/γ-CDngs	22.4
